# Whole-genome sequencing-based antimicrobial resistance and shedding dynamics of *Escherichia coli* isolated from calves before and after antimicrobial group treatments

**DOI:** 10.1128/spectrum.03214-23

**Published:** 2024-03-05

**Authors:** Véronique Bernier Gosselin, Javier E. Fernandez, Catherine Ollagnier, Isabelle Morel, Raphael Siegenthaler, Alexandra Collaud, Mireille Meylan, Vincent Perreten

**Affiliations:** 1 Clinic for Ruminants, Vetsuisse Faculty, University of Bern, Bern, Switzerland; 2 Division of Molecular Bacterial Epidemiology and Infectious Diseases, Institute of Veterinary Bacteriology, Vetsuisse Faculty, University of Bern, Bern, Switzerland; 3 Pigs, Agroscope, Posieux, Switzerland; 4 Ruminant Nutrition and Emissions, Agroscope, Posieux, Switzerland; 5 Research Contracts Animals, Agroscope, Posieux, Switzerland; Innovations Therapeutiques et Resistances, Toulouse, France

**Keywords:** *Escherichia coli*, fecal carriage, antimicrobial resistance, molecular epidemiology, cattle

## Abstract

The fattening of calves is often associated with high antimicrobial use and the selection of antimicrobial resistance (AMR). The objective of this observational longitudinal study was to describe the AMR and strain dynamics, using whole-genome sequencing (WGS), of fecal *Escherichia coli* in a cohort of 22 calves. All calves received antimicrobial group treatments on Day (D) 1 (oxytetracycline, intramuscularly) and on D4 through D12 (doxycycline, in-feed). Additionally, eight calves received individual parenteral treatments between D7 and D59, including florfenicol, amoxicillin, marbofloxacin, and gamithromycin. Rectal swabs were collected from all calves on D1 (prior to treatment), D2, D9, and D82. The swabs were spread onto *Enterobacterales*-selective agar, and three *E. coli* colonies per plate were subjected to WGS. Out of 264 isolates across all calves and sampling times, 80 unique strains were identified, a majority of which harbored genes conferring resistance to tetracyclines, streptomycin, and sulfonamides. The diversity of strains decreased during the in-feed antimicrobial group treatment of the calves. On D82, 90% of isolates were strains that were not isolated at previous sampling times, and the median number per strain of AMR determinants to tetracyclines, florfenicol, β-lactams, quinolones, or macrolides decreased compared to D9. Additionally, clonal dissemination of some strains represented the main transmission route of AMR determinants. In this study, WGS revealed important variations in strain diversity and genotypic AMR of fecal *E. coli* over time in calves subjected to group antimicrobial treatments.

**IMPORTANCE:**

The continued emergence and spread of antimicrobial resistance (AMR) determinants are serious global concerns. The dynamics of AMR spread and persistence in bacterial and animal host populations are complex and not solely driven by antimicrobial selection pressure. In calf fattening, both antimicrobial use and carriage prevalence of antimicrobial-resistant bacteria are generally recognized as high. This study provides new insights into the short-term, within-farm dynamics and transmission of AMR determinants in *Escherichia coli* from the dominant fecal flora of calves subjected to antimicrobial group treatments during the rearing period. The diversity of *E. coli* strains decreased over time, although, in contrast to previous observations in extended-spectrum β-lactamase-producing *Enterobacterales*, the predominance of a few clones was not observed. The spread of AMR determinants occurred through the dissemination of clonal strains among calves. The median number per strain of AMR determinants conferring resistance to selected antimicrobials decreased toward the end of the rearing period.

## INTRODUCTION

In most countries, the fattening of veal calves involves the transport and commingling of calves from multiple dairy farms of origin to the fattening facility. There may be variations between countries with regard to feeding, housing conditions, and duration of the fattening period. In Switzerland, calves are purchased around the age of 30 days, housed in groups, fed mainly milk or milk substitute, with constant free access to water and roughage, and slaughtered around the age of 160 days (1). The commingling of large numbers of calves from multiple origins and of various health and immune status is a risk factor for increased morbidity and mortality (1, 2). Thus, group treatment with antimicrobials administered via feed or parenteral injections at the beginning of the fattening period is common practice and results in high antimicrobial use (AMU) (3–5). In Switzerland, antimicrobial prescription data for 2020 revealed that fattening cattle was the animal category that was attributed the highest rank in terms of the amount of active substance prescribed, and the second highest rank in terms of the number of treatments (6). In turn, AMU in general, as well as group or oral treatment specifically, has been associated with increased antimicrobial resistance (AMR) in commensal and pathogenic bacteria in veal calves (3, 7, 8).

The dynamics of AMR in bacteria isolated from veal calves are complex. In investigation reports on the dynamics of phenotypic AMR over the course of the fattening period, changes in AMR varied according to the AMR indices used, bacterial species, and study farms. For instance, based on the antimicrobial drug concentration inhibiting the growth of 50% of tested isolates (MIC_50_), AMR increased among *Escherichia coli* (for azithromycin) and *Mannheimia haemolytica* isolates (for clindamycin and tiamulin) (9). In another study from arrival to departure from the farm, in which 98% of treatments were group treatments, the proportion of *E. coli* isolates resistant to amoxicillin, tetracyclines, streptomycin, and sulfonamides increased, whereas the proportion of isolates resistant to β-lactams other than amoxicillin and to quinolones decreased (10). A reduction in other AMR indices, such as the antimicrobial resistance index (the proportion of tested drugs to which an isolate shows resistance) or the proportion of multidrug-resistant isolates (fecal *E. coli*, nasal *Pasteurella multocida,* and *M. haemolytica*), through the fattening period was also reported (3, 9). In other studies with a focus on extended-spectrum β-lactamase (ESBL)-producing *Enterobacterales*, the AMR dynamics in veal calf populations were investigated using strain typing of isolates [based on either pulsed-field gel electrophoresis (PFGE), multilocus sequence typing (MLST), or a combination of ESBL gene sequence, MLST, and plasmid replicon typing]. Upon arrival at the fattening farms, a diversity of isolates was observed, which was attributed to the multiple farms of origin of the calves (10, 11). Some clonal variants were detected at subsequent sampling time points that were not detected upon arrival, which could be explained by their clonal expansion following antimicrobial group treatment or by their source being the fattening farm environment (11, 12). Over the fattening period, the diversity decreased, and the dominance of a limited number of clones was observed. These results, along with those of studies on dairy farms, suggest that the dynamics of ESBL gene dissemination are the result of a combination of the clonal spread of resistant strains, horizontal transfer of plasmids between different *E. coli* strains, and transfer of genes between plasmids (10, 11, 13–15). Mathematical modeling of ESBL carriage (determined phenotypically) further supported that both between-animal transmission and sporadic introduction affect AMR dynamics, whereas AMU affects the clearance of the ESBL phenotype rather than its acquisition (16). Nevertheless, it was previously pointed out that the phenotypic AMR dynamics observed among the subpopulation of ESBL-producing isolates do not necessarily reflect those of the dominant *E. coli* flora (isolated on non-selective medium) (10, 17), and this might also be the case for genotypic AMR dynamics.

As aforementioned, previous reports on the AMR dynamics of the dominant *E. coli* population in calves over the course of the fattening period were mainly based on phenotypic AMR, which provides limited information on the strain dynamics underlying the observed phenotypes. Therefore, the objective of this study was to describe the dynamics of whole-genome sequencing (WGS)-based AMR in commensal fecal *E. coli* isolated from a cohort of calves subjected to antimicrobial group treatments.

## RESULTS

### Study population

Twenty-two calves were studied longitudinally from arrival at a research facility (D0), where they were housed with 22 other calves, until the end of the rearing period (week 13). The calves were weaned at week 7. The median (range) age and weight of the study calves upon arrival were 27 (19–72) days and 75 (55–89) kg. All animals were crossbreds of a Brown Swiss dam and a Simmental (*n* = 11), Limousin (*n* = 7), or Angus (*n* = 4) sire. On D1 (before any treatment), D2, D9, and D82, rectal swab samples were collected from each calf. All calves received antimicrobial group treatments consisting of long-acting oxytetracycline intramuscularly on D1, followed by in-feed doxycycline from D4 to D12. Additionally, over the study period, eight calves received individual systemic treatments after a clinical diagnosis of infectious bronchopneumonia, including two calves treated twice each with different drugs (Table 1). The antimicrobial drugs used included florfenicol (*n* = 5 calves), amoxicillin (*n* = 2), marbofloxacin (*n* = 2), and gamithromycin (*n* = 1). One individual treatment occurred between the second (D2) and third (D9) samplings, whereas the remaining individual treatments occurred after the third sampling (D9) and a minimum of 23 days prior to the fourth sampling (D82).

**TABLE 1:** Individual calf antimicrobial treatments

Day(s) of treatment	Calf identification number	Antimicrobial drug	Administration route^*a*^	Recommended dosing interval^*b*^
D7–10	604	Marbofloxacin	IM	Once daily for 3 days
D13 + D15	621	Amoxicillin	IM	Twice at 48-h interval
D16 + D18	610 and 616	Florfenicol	IM	Twice at 48-h interval
D17 + D19	618^*c*^	Florfenicol	IM	Twice at 48-h interval
D17 + D19	607^*c*^	Amoxicillin	IM	Twice at 48-h interval
D21	614	Gamithromycin	SC	Once
D22	607^*c*^	Florfenicol	IM	Twice at 48-h interval
D28–30	618^*c*^	Marbofloxacin	IM	Once daily for 3 days
D55 + D57+ D59	605	Florfenicol	IM	Twice at 48-h interval

^*a*^ IM, intramuscular and SC, subcutaneous.

^*b*^ Swiss Compendium of Veterinary Products (Institut für Veterinärpharmakologie und –toxikologie. Tierarzneimittelkompendium der Schweiz, Zurich, Switzerland. https://www.vetpharm.uzh.ch/tak/. Accessed 27 July 2023).

^*c*^ Two calves each treated twice.

### Strains

**Fig 1:**
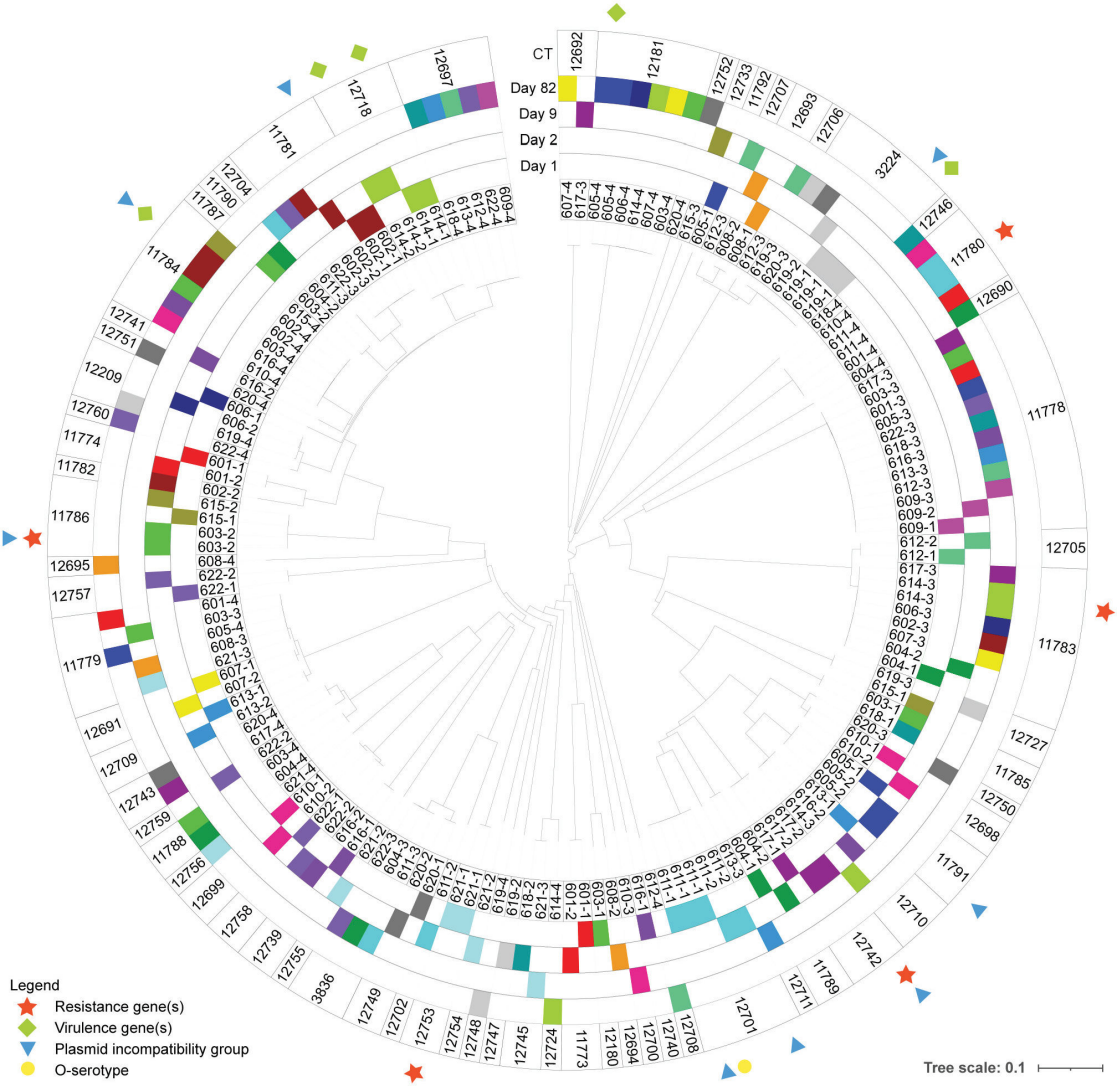
Phylogenetic tree representing the genetic relationship between the 155 *Escherichia coli* isolated from calves, based on core genome MLST complex type (CT; outer circle). Isolate numbers (inner circle) are composed of the calf ID and sampling time, respectively. Each color represents a calf, and the position of each colored band represents the sampling time at which the strain was isolated. Within a CT, when non-identical within-sample isolates (same calf and time) are shown, their difference is indicated as the presence or absence of resistance genes (star), virulence genes (diamond), plasmid incompatibility group (triangle), or O type (circle).

From 88 rectal swab samples, a total of 264 *E. coli* isolates (three per sample) and their respective genome sequences were obtained (Table S1). In 35 samples, all three isolates were identical [i.e., three isolates with identical core genome MLST complex type (CT), *in silico* serotype, AMR and virulence genes, and plasmid incompatibility groups within the same calf and time]. Those were distributed over time as 8, 8, 11, and 8 samples on D1, D2, D9, and D82, respectively. In 39 samples (12, 9, 7, and 11 samples on D1, D2, D9, and D82, respectively), two different strains were isolated, whereas 14 samples (2, 5, 4, and 3 samples on D1, D2, D9, and D82, respectively) yielded three different strains. After the removal of within-sample replicates, 155 isolates remained (38, 41, 37, and 39 isolates from D1, D2, D9, and 82, respectively), represented in a phylogenetic tree in Fig. 1. Across all calves and sampling times, 80 unique strains (unique set of CT, AMR, and virulence genes) were obtained. Of those, 39 were isolated each from one single sample, whereas the remaining had clones isolated from 2 up to 11 samples. The 80 unique strains belonged to 39 sequence types (ST), the most common being ST58 and ST10. The detailed distribution of strains is shown in Table 2. The sequence types ST13057, ST13058, and ST13059 represented newly assigned STs.

**TABLE 2:** Distribution of sequence type and core genome multi-locus sequence typing based CT among the 80 unique *E. coli* strains isolated from 22 calves^*a*^

Sequence type	Number of strains	CT
58	15	11781, 11784, 11787, 11790, 12209, 12697, 12704, 12718, 12751
10	10	11785, 11789, 12698, 12701, 12711, 12742, 12750
69	5	3224, 12693, 12706, 12707
2522	4	11779, 12691, 12709
162	3	3836, 12702, 12749
301	3	11783, 12705
446	3	11788, 12743, 12759
101	2	12699, 12758
329	2	11780
8580	2	12181
10850	2	11791, 12710
13057	2	12753

^*a*^ The remaining ST were represented by a single strain: ST21 (CT 12745), ST56 (CT 11786), ST57 (CT 12692), ST117 (CT 11792), ST120 (CT 12740), ST124 (CT 12755), ST155 (CT 11774), ST306 (CT 12700), ST339 (CT 12746), ST361 (CT 11778), ST410 (CT 12180), ST448 (CT 12748), ST675 (CT 12739), ST718 (CT 12756), ST723 (CT 12724), ST730 (CT 12690), ST783 (CT 11773), ST949 (CT 11782), ST1302 (CT 12727), ST1308 (CT 12754), ST2521 (CT 12757), ST3057 (CT 12752), ST3249 (CT 12694), ST3695 (CT 12695), ST6126 (CT 12708), ST13058 (CT 12733), and ST13059 (CT 12747).

### AMR determinants

A majority of the 80 strains harbored genes conferring resistance to tetracyclines (*n* = 57), streptomycin (*n* = 49), and sulfonamides (*n* = 42). The 80 strains also harbored genes conferring resistance to β-lactams (*n* = 38), kanamycin (*n* = 22), trimethoprim (*n* = 19), chloramphenicol (*n* = 19), florfenicol (*n* = 15), quinolones (*n* = 11), gentamicin (*n* = 10), erythromycin/telithromycin/tylosin (*n* = 6), tobramycin (*n* = 4), hygromycin (*n* = 3), fosfomycin (*n* = 3), and lincosamides (*n* = 1). The most frequent plasmid incompatibility groups were IncFIB (AP001918) (*n* = 38 strains), IncQ1 (*n* = 28), and IncI1-Iα (*n* = 24). Up to six incompatibility groups per strain were detected. In 16 unique strains, no known plasmid incompatibility group was detected, including strains such as those belonging to CT 11778 (ST361) and 12692 (ST57) harboring numerous AMR genes.

### Dynamics of AMR determinants over time

**Fig 2:**
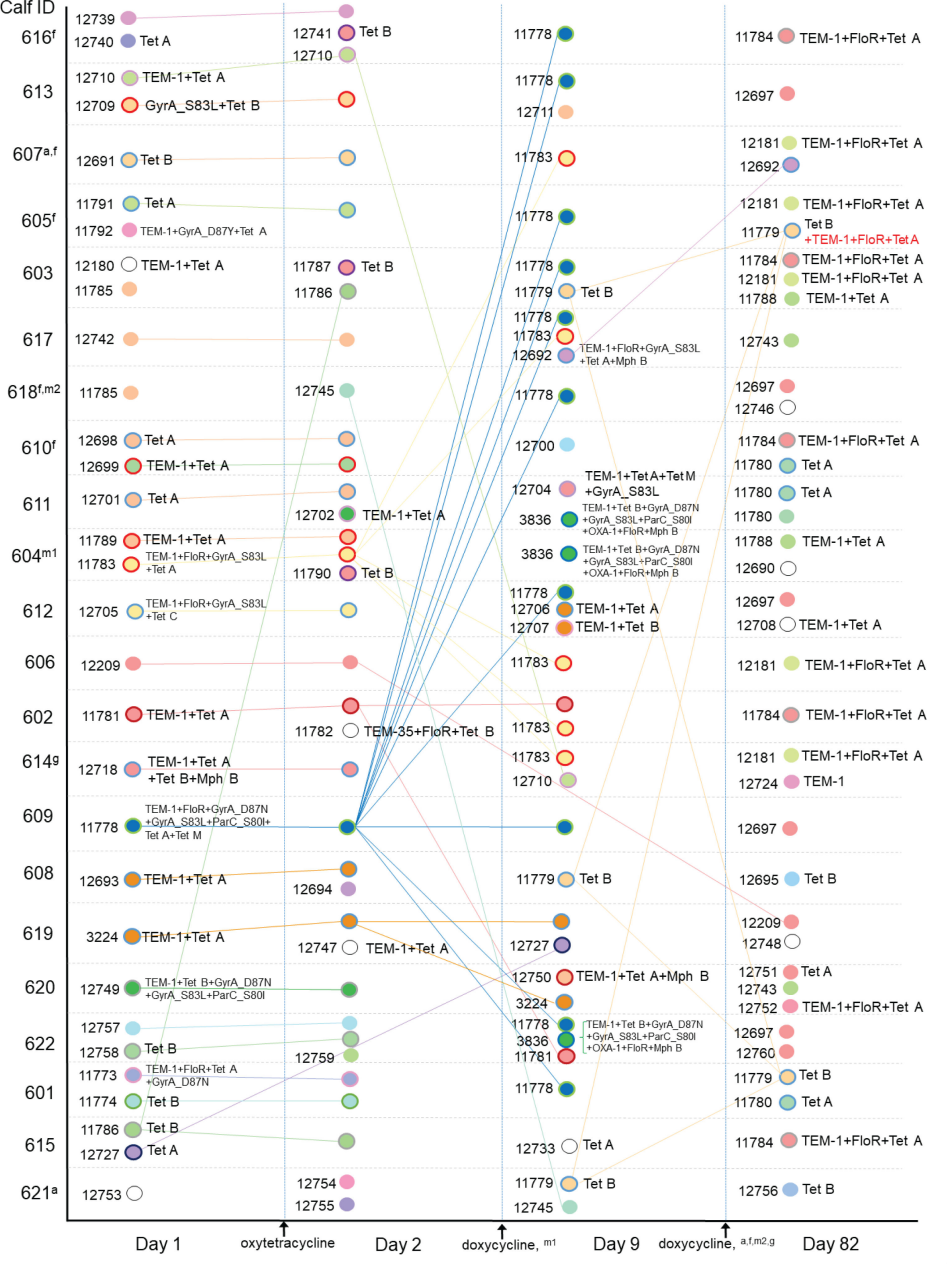
Distribution among calves and dynamics over time of *Escherichia coli* isolates, with respective core genome MLST CT, sequence type (inner circle color), and determinants of resistance to antimicrobial drugs used during the study (outer circle color). For instance, on D1, calves 606, 602, and 614 carried isolates of different CT belonging to ST58 (same inner circle color) and harbored different resistance determinants’ combinations (different outer circle colors) or lack thereof. In cases of within-sample isolates with identical CT and shown resistance determinants (clonal or not), a single one of the strains is shown for improved clarity. Resistance determinants written in red represent an addition to the temporally previously observed combination of CT and resistance determinants. Connecting lines between isolates at different time points indicate clonal isolates based on CT. Superscripts indicate which calves and at which time interval between samplings received individual antimicrobial treatments: amoxicillin (a), florfenicol (f), gamithromycin (g), and marbofloxacin (m; first and second treatment incidence).

**Fig 3:**
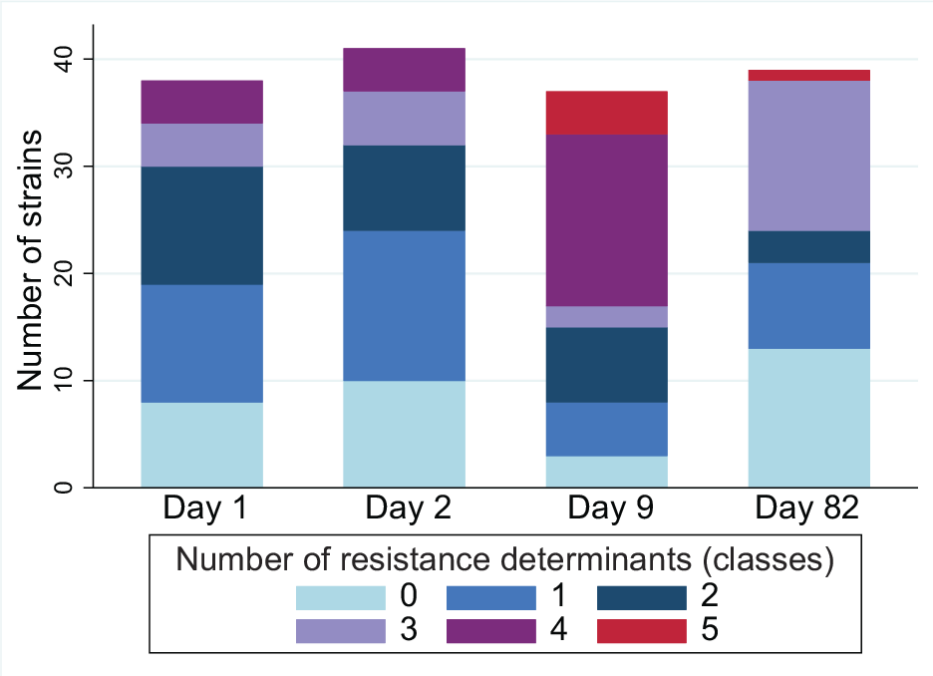
Distribution of the number of *E. coli* strains according to the number of resistance determinants they harbored against different antimicrobial drugs or classes (tetracyclines, florfenicol, β-lactams, quinolones, and macrolides) at four sampling times. Within a strain, multiple determinants against a single class were counted as one. After the removal of within-sample replicates, the total number of isolates on D1, D2, D9, and D82 was 38, 41, 37, and 39, respectively.

**Fig 4:**
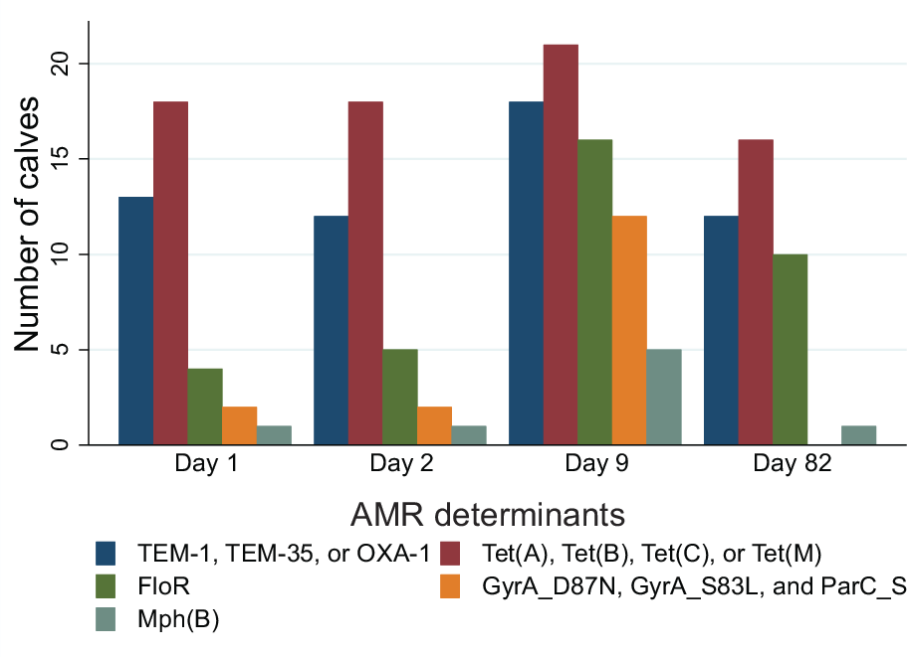
Distribution of the number of calves carrying at least one *E. coli* harboring selected AMR determinants or combination thereof, at four sampling times. Antibiotic resistance determinants and their function: GyrA_D87N, DNA gyrase subunit A with substitution of aspartic acid (D) to asparagine (N) at codon 87 for quinolone resistance; GyrA_S83L, DNA gyrase subunit A with substitution of serine (S) to leucine (L) at codon 83 for quinolone resistance; ParC_S80I, DNA topoisomerase 4 subunit A with substitution of serine (S) to isoleucine (I) at codon 80 for quinolone resistance; Tet(A), Tet(B), and Tet(C), tetracycline efflux; Tet(M), ribosomal protection for tetracycline resistance; OXA-1, TEM-1, and TEM-35, β-lactamase; FloR, phenicol efflux; and Mph(B), macrolide phosphotransferase.

The distribution of strains per calf and sampling time was determined based on cgMLST phylogenetic relationship analysis and their AMR determinants to antimicrobial drugs or classes used for group or individual treatments during the study (tetracyclines, florfenicol, β-lactams, quinolones, and macrolides; Fig. 2). Among calves, the most prevalent AMR determinants were those conferring resistance to β-lactams, tetracyclines, florfenicol, and quinolones, including TEM-1, Tet(A), FloR, GyrA_S83L, and Tet(B), detected at least once in 21, 21, 19, 17, and 16 calves, respectively. The distribution of AMR determinant combinations among the 80 unique strains is shown in Table 3. Among the strains isolated at each sampling time, the distribution of the number of antimicrobial drugs or classes (tetracyclines, florfenicol, β-lactams, quinolones, and macrolides) against which a strain carried at least one resistance determinant [e.g., a combination of Tet(A) and Tet(B) counted as 1] is shown in Fig. 3. The median number of drugs or classes was 1.5, 1, 4, and 1 on D1, D2, D9, and D82, respectively. The distribution of selected AMR determinants among calves over time is illustrated in Fig. 4. The number of calves carrying *E. coli* strains with the combination of GyrA_D87N, GyrA_S83L, and ParC_S80I increased from two calves on D1 and D2 to 12 calves on D9 (including one calf treated with marbofloxacin between D2 and D9), then decreased to 0 on D82. Similarly, the number of calves carrying *E. coli* containing FloR increased from 4 and 5 calves on D1 and D2, respectively, to 16 and 10 calves on D9 and D82, respectively. Of note, the carriage of *E. coli* containing FloR increased before the incidence of any individual calf treatment with florfenicol (i.e., between D9 and D82). On D82, four of the five florfenicol-treated calves had *E. coli* carrying FloR, three of which already had strains carrying FloR on D9 (but of different STs). However, FloR carriage was also common among calves not directly exposed to florfenicol (6 out of 17 calves on D82). The carriage prevalence of the remaining AMR determinants of interest was relatively stable over time.

**TABLE 3:** Distribution of determinants of antimicrobial resistance to tetracyclines, florfenicol, β-lactams, quinolones, and macrolides, alone or in combination, with corresponding sequence types, among 80 unique *E. coli* strains isolated from 22 calves

AMR determinant^*a*^ combinations	*n*	Sequence types
Tet(A)	10	10 (*n* = 4), 58, 120, 329, 1302, 10850, and 13058
Tet(B)	10	56, 58 (*n* = 3), 101, 718, 2522 (*n* = 2), 3695, and 155
Tet(B) + GyrA_S83L	1	2522
TEM-1	1	723
TEM-1 + Tet(A)	13	10, 58, 69 (*n* = 4), 101, 162, 410, 446, 6126, 10850, and 13059
TEM-1 + Tet(B)	1	69
TEM-1 + Tet(A) + Mph(B)	1	10
TEM-1 + Tet(A) + Tet(B) + Mph(B)	3	58 (*n* = 3)
TEM-1 + Tet(A) + FloR	6	3057, 8580 (*n* = 2), and 58 (*n* = 3)
TEM-1 + Tet(A) + Tet(B) + FloR	1	2522
TEM-35 + Tet(B) + FloR	1	949
TEM-1 + Tet(A) + FloR + GyrA_S83L	2	301 (*n* = 2)
TEM-1 + Tet(A) + FloR + GyrA_S83L + Mph(B)	1	57
TEM-1 + Tet(C) + FloR + GyrA_S83L	1	301
TEM-1 + Tet(A) + FloR + GyrA_D87Y	1	783
TEM-1 + Tet(A) + GyrA_D87Y	1	117
TEM-1 + Tet(A) + Tet(M) + GyrA_S83L	1	58
TEM-1 + Tet(B) + GyrA_D87N + GyrA_S83L + ParC_S80I	1	162
TEM-1 + Tet(A) + Tet(M) +FloR + GyrA_D87N + GyrA_S83L + ParC_S80I	1	361
OXA-1 +TEM-1 +Tet(B) +FloR + Mph(B) +GyrA_D87N + GyrA_S83L + ParC_S80I	1	162

^*a*^ Antibiotic resistance determinants and their function: GyrA_D87N and GyrA_D87Y, DNA gyrase subunit A with substitution of aspartic acid (D) to asparagine (N) or to tyrosine (Y) at codon 87 for quinolone resistance; GyrA_S83L, DNA gyrase subunit A with substitution of serine (S) to leucine (L) at codon 83 for quinolone resistance; ParC_S80I, DNA topoisomerase 4 subunit A with substitution of serine (S) to isoleucine (I) at codon 80 for quinolone resistance; Tet(A), Tet(B), and Tet(C), tetracycline efflux; Tet(M), ribosomal protection for tetracycline resistance; OXA-1, TEM-1, and TEM-35, β-lactamase; FloR, phenicol efflux; and Mph(B), macrolide phosphotransferase.

### Diversity and dissemination of *E. coli* strains

Overall, there was a high level of similarity between *E. coli* strains from D1 and D2 samplings, where isolates from D2 were clones of isolates from D1 in 19 calves (as indicated by connecting lines in Fig. 2). Conversely, only 4 out of 39 strains from D82 were clones of isolates identified at previous sampling times. The diversity of strains varied over time. On D1 and D2, 34 and 37 unique strains were identified, belonging to 20 and 21 STs, respectively. At each of these samplings, a single one of these strains [CT 11785 (ST10) on D1 and CT 11786 (ST56) on D2] had a clone also isolated from a different calf. At samplings on D9 and D82, 18 unique strains (13 STs) and 24 unique strains (14 STs), respectively, were identified, and 19 and 15 clones of these strains (all strains combined), respectively, were simultaneously found in other calves. Dispersion of some of the clones among calves is illustrated in Fig. 1 and 2. For instance, two strains [CT 11778 (ST361) and CT 11783 (ST301)] carrying resistance determinants to tetracyclines, β-lactams, florfenicol, and quinolones were initially isolated from a single calf (ID 609 and 604, respectively) at D1 and D2, and from 10 and 6 calves, respectively, on D9. Together, these two strains were the main drivers (16/21 strains) for the increased prevalence of florfenicol and quinolone resistance determinant carriage on D9. However, neither of these clones were detected in any calf on D82. Also noticeable in Fig. 2 is the fact that a combination of AMR determinants, TEM-1, FloR, and Tet(A), appeared on D82 in multiple calves harboring clones that were not observed at previous samplings [CT 11784 (ST58) and CT 12181 (ST8580)]. On the other hand, the clonal spread of strains without AMR determinants was also observed, such as strains of CT 12697 (ST58) detected in five calves on D82.

Of 36 groups of clonal isolates (based on identical CT) that were either repeatedly isolated in a calf over time or shared between calves or both, the isolates were identical within group for 26 CT, whereas the isolates belonging to 10 CT differed within CT with regards to AMR genes (*n* = 5), virulence genes (*n* = 4), or O-serotype (*n* = 1). One isolate [CT 11779 (ST2522)] at D82 carried the AMR determinants TEM-1, FloR, and Tet(A), which were absent from the other strains of CT 11779 (which all were identical clones) isolated on D9 and D82. Inspection of plasmid incompatibility groups by PlasmidFinder revealed that this isolate carried a plasmid IncI1-Iα, whereas no plasmid incompatibility group was detected in other CT 11779 clones. The strain was further investigated by comparing its pangenome with that of another CT 11779 isolate from D9. Acquired singleton gene clusters, representing the acquired material that differed between the two strains, were extracted as concatenated DNA and compared with the GenBank database using the nucleotide-BLAST algorithm (www.ncbi.nlm.nih.gov). There was a 100% similarity with the sequence of an *E. coli* plasmid p1514kTc1_N_I1_ST113 from the database (GenBank accession no. NZ_MW800641), indicating the acquisition of a plasmid. To investigate whether the AMR genes acquired by this strain were located on a plasmid, the contigs from the assembly were aligned with the plasmid p1514kTc1_N_I1_ST113 using Mauve (option MCM). As a result, determinants of AMR to β-lactams (TEM-1), aminoglycosides [APH(3″)-Ib and APH(6)-Id], and sulfonamides (Sul2) were identified, flanked by the insertion sequence IS26 (associated with the mobilization of AMR genes), along with the plasmid replication initiation protein RepZ (associated with IncI plasmids). Thus, the acquisition of these AMR determinants was confirmed to be associated with the acquisition of a plasmid.

## DISCUSSION

The objectives of this study were to describe the AMR dynamics in 264 commensal fecal *E. coli* isolates collected longitudinally from calves in the rearing period subjected to antimicrobial group treatments and to characterize them using WGS for the identification of AMR genes and strain typing. Already upon arrival, a majority of calves (18/22) harbored *E. coli* carrying AMR genes. Genotypic AMR to tetracyclines, β-lactams, streptomycin, and sulfonamides, which are antimicrobial classes commonly used in cattle, was predominant among the study calves and among the collection of unique strains. This population of resistant strains is likely well established in calves, possibly belonging to the normal flora, and maintained through frequent AMU. This finding is similar to previous reports of phenotypic AMR in commensal *E. coli* in veal calves in Switzerland, France, and Belgium (3, 9, 10). In these previous studies, variations in the proportions of isolates showing phenotypic AMR between two different time points were observed. In the present study, variations in the number of calves carrying isolates with specific AMR determinants were also observed. The larger number of sampling times than in aforementioned studies (four vs two) provided additional detail, for instance, the observed initial increase in the number of calves carrying isolates with quinolone resistance determinants, followed by a decrease at the last sampling time, namely 82 and 81 days after the calves’ arrival to the research facility and the first antimicrobial treatment, respectively. However, it cannot be excluded that short-term changes in AMR carriage between sampling times could have been missed. Moreover, the selection of three colonies per sample, rather than a single one as in the aforementioned studies, allowed to capture a wider diversity of *E. coli* strains, within samples and overall.

The overall diversity of *E. coli* strains varied over time (range of 18–37 unique strains per sampling time). A large diversity of strains was observed upon arrival, consistent with previous reports and likely attributable to the diverse origins of the calves (10, 11). There was limited variation between D1 and D2, which would suggest that the level and duration of exposure of the fecal flora to oxytetracycline administered via parenteral injection to the calves between these two samplings were insufficient to elicit a major shift in the dominant flora. In contrast, on D9 (i.e., 5 days after the start of in-feed doxycycline treatment), the number of unique strains decreased, and the number of clonal strains shared among calves increased. In studies on ampicillin-resistant *E. coli* in beef calf cohorts (not subjected to antimicrobial group treatment), the predominant genotypes (assessed by PFGE fingerprinting) showed temporal variations, but the number of different genotypes at each sampling remained relatively stable (18, 19). Based on the dynamics of phenotypic AMR profiles, Catry et al. (3) suggested that oral antimicrobial group treatment in veal calves results in the selection of a large number of resistant strains rather than a small number of resistant clones. This was partially supported by our findings based on WGS. In contrast, in studies on ESBL-producing *E. coli* subpopulations in veal calves, strain typing revealed an important reduction in strain diversity and the dominance of a few clones at the end of the study period (10 weeks and 5 months, respectively) (10, 11). This contrast suggests that AMR dynamics may differ, not only based on the diversity of animal sources and antimicrobial treatment but also depending on whether the bacterial populations of interest are *E. coli* from the dominant flora or the ESBL-producing subdominant flora.

An additional observation in the present study was that only 10% of isolates on D82 were clones of previously identified strains. Similar observations have been reported for ESBL-producing strains in veal calves (11). Some strains may have initially been part of the calves’ subdominant flora but gone undetected due to the isolate selection strategy since only three colonies per plate were selected. Other strains may have been acquired from the barn environment. These previously undetected strains carried on average fewer AMR determinants than the strains isolated on D9, which they had displaced or replaced (1.56 vs 2.95 AMR determinants).

In previous studies with a focus on ESBL-producing *Enterobacterales*, the spread of AMR genes was reported to occur through clonal dissemination of strains, although horizontal transfer of plasmids between strains and of genes between plasmids was also suspected (10–13). In the present study, strains were not isolated using a medium selecting specific resistant strains such as ESBL-producing *Enterobacterales,* but AMR determinants in the dominant *E. coli* fecal flora also appeared to spread among calves predominantly by clonal strains. The concurrent acquisition of a plasmid and AMR genes was suspected in one isolate of CT 11779, although not all of the acquired AMR genes could be confirmed to be plasmid associated. However, it cannot be excluded that the transfer of plasmids and associated AMR genes also occurred between clones of different CT. Further studies using a more systematic, in-depth characterization of isolates with long-reads WGS may provide supporting evidence for this AMR determinant transmission pathway. Such genomic characterization has allowed the identification of different patterns of AMR gene dissemination dynamics between dairy farms in a previous study (15). The generalizability of the present study’s findings regarding AMR determinant transmission dynamics is limited by the inclusion of a single-rearing facility and cohort. Indeed, based on a high genetic diversity of ESBL-producing *E. coli* strains from slaughtered animals, other authors postulated that the spread of ESBL genes among *E. coli* in animal populations occurs either by plasmids or by a diversity of pathways (17, 20). This means that different modes of transmission of AMR determinants may concomitantly be at play, and the predominant mode may vary according to the studied bacterial population and host population (e.g., at herd or national level).

Antimicrobial use is recognized as a major driver for AMR, and the association of antimicrobial group treatments with decreased phenotypic antimicrobial susceptibility in commensal *E. coli* in veal and dairy calves was reported in previous studies (8, 21). In the present study, all calves were treated parenterally with oxytetracycline and orally with doxycycline, and a third (8/22) of calves were additionally treated with a variety of other antimicrobial drugs. Tetracycline resistance determinants were present in numerous strains among different calves already prior to treatment, and the selection pressure of treatment may have contributed to their maintenance in the *E. coli* population until D82. Additionally, the proportion of calves carrying isolates harboring florfenicol or quinolone resistance determinants increased over time, including prior to florfenicol treatments and in calves that were not treated with these antimicrobial classes. Similarly, in a previous study in stocker calves receiving macrolide metaphylaxis, the proportion of quinolone-resistant *M. haemolytica* increased 7 days after treatment, compared to pre-treatment (22). In another study in preweaned calves, those receiving macrolide metaphylaxis had an increased proportion of quinolone-resistant *E. coli* compared to untreated calves from day 2 to day 7 after treatment (23). A possible explanation for these observations was co-selection, where the use of an antimicrobial drug results in the selection of resistance to drugs of different classes. In the present study, all isolates carrying florfenicol or quinolone resistance determinants also carried tetracycline resistance determinants and showed an increase in prevalence, which could be the result of co-selection following oxytetracycline and doxycycline treatments. Regarding the impact of individual calf treatments on AMR carriage or maintenance, a clear trend was neither identified among calves that received additional individual treatments nor in other calves in this study.

A strength of this longitudinal study is that multiple sampling points over an extended time period were included. However, due to the large time gap between the last day of group treatment (D12), or individual treatments where applicable, and the next sample collection (D82), short-term changes in AMR carriage during that period might have been missed. Additionally, three colonies per fecal sample culture (morphologically different whenever possible) were selected for characterization, which increased the total number of different strains to 155 (compared to 88 if a single isolate per sample had been selected). This selection method provided a measure of within-sample strain diversity as it revealed that ≥2 strains could be retrieved from a majority (53/88) of samples. The optimal number of isolates needed to capture the full spectrum of *E. coli* strains in the calves’ flora is unknown. In a previous study where 20 colonies per swab were characterized based on a combination of phenotypic AMR pattern, biotype (based on fermentation pattern), and O-serogroup (where possible), an overall mean of 5.17 strains per swab was reported (24). Although it is conceivable that the selection of five or six isolates per sample in the present study could have resulted in a larger diversity of strains, the proportion of replicate strains would likely also have been higher. Nevertheless, the selection of three isolates allowed for the detection of variations in strain diversity and in their clonal spread during and after a course of antimicrobial treatment.

In this observational study, fecal samples were longitudinally collected from a group of calves reared in conditions reflecting those of Swiss veal-fattening facilities (e.g., multiple origins, group housing, and antimicrobial group treatment). In the calf fattening industry, antimicrobial group treatments represent a major portion of AMU and are therefore a key target for reducing AMU (2–5). Strain-typing data from the present study suggest that the clonal spread of commensal fecal *E. coli* easily occurs among commingled calves. In addition to the spread of AMR determinants by clonal strains, the location of AMR determinants on mobile genetic elements such as plasmids facilitates their transfer between bacteria, including potential transfer from commensal to pathogenic and zoonotic bacteria. With regards to calf health, whether AMR selection and their maintenance in commensal bacteria have a negative impact on response to therapeutic antimicrobial treatment of diseases caused by pathogenic bacteria remains to be determined (22).

In conclusion, in this cohort of calves subjected to antimicrobial group treatments, analysis of the genotypic AMR dynamics among commensal fecal *E. coli* isolates revealed a reduction in strain diversity during treatment. Furthermore, clonal dissemination of strains among calves was the primary mode for the spread of AMR determinants, although the acquisition of plasmid-mediated AMR genes by a strain was also suspected. Despite the rapid initial clonal expansion of some strains, their replacement by other strains by the time of the last sampling led to a reduction in carriage prevalence for some AMR determinants, while others were maintained, underlining the complexity of AMR dynamics in the dominant fecal *E. coli* flora of calves during the rearing period.

## MATERIALS AND METHODS

### Animals and sample collection

This was an observational longitudinal study. Twenty-two calves were gathered for rearing in a research facility in Switzerland. A total of 8 calves first transited through a livestock market, and the remaining 14 calves were collected from 12 different farms of origin and transported together. Upon arrival, the calves were examined for clinical signs of disease, weighed, and assigned unique identification numbers. Four calves had diarrhea (undetermined cause). The calves were then housed together in one pen, which had been previously cleaned and disinfected, and remained in this pen for the entire study period. In the same barn were housed 22 other calves that were delivered the following day (but not included in the present study), 5 of which had fever upon clinical examination at arrival. The calves were fed milk replacer through an automatic distribution system. The daily amount of milk replacer was gradually increased from 4 L upon arrival to 6 L at 3 weeks after arrival, then gradually decreased until weaning, at 7 weeks after arrival. Concentrates, hay, and corn silage were offered from week 1, week 2, and week 5, respectively. The calves were reared over 13 weeks until they reached 160 kg on average. Upon arrival, all calves received iron (563 mg/calf, orally) and selenium (1.6 mg/calf, subcutaneously) supplementation. On the day after arrival (D1), all study calves received metaphylactic antimicrobial treatment with long-acting oxytetracycline, 20 mg/kg of body weight, intramuscularly. From D4 to D12, the calves received doxycycline in milk replacer at a daily rate of 17.5 mg/kg of body weight. Any additional antimicrobial treatment of individual calves during the rearing period was administered at the discretion of the treating veterinarian and was recorded. Rectal swab samples were collected on D1 (before the first treatment), D2 (after intramuscular antibiotic treatment), D9 (during oral antibiotic treatment), and D82 (end of the rearing period). The swabs (TRANSWAB Gel Amies Plain, mwe, UK) were transported at room temperature in Amies medium to the laboratory for processing within 4 hours. The sampling period spanned from February to April 2020.

### Bacterial culture and species identification

The swabs were spread directly onto *Enterobacterales*-selective BROLAC agar (Thermo Fisher Scientific, Waltham, USA), and the plates were incubated at 37°C for 24 hours. Three lactose-fermenting colonies exhibiting a dissimilar morphology were selected per plate to take the within-sample strain diversity into account. If no difference in morphology was observed, the colonies were chosen randomly. Species identification was assigned using matrix-assisted laser desorption-ionization time-of-flight mass spectrometry (Microflex LT, Bruker Daltonics GmbH, Bremen, Germany). If one of the selected colonies was not identified as *E. coli* (in less than 10 instances), an additional colony was selected in order to obtain a total of three *E. coli* per sample. Isolates identified as *E. coli* were regrown in pure culture on trypticase soy agar containing 5% sheep blood (TSA-SB; Becton, Dickinson and Company, NJ, USA) and stored in 30% glycerol at −80°C.

### Whole-genome sequencing data

For library preparation, the isolates were grown on TSA-SB, and bacterial DNA was extracted using proteinase K and mechanical disruption using glass beads (PowerBead, Qiagen, Hilden, Germany). The extracted DNA was purified using the AMPure XP paramagnetic bead-based chemistry (Beckman Coulter, Brea, CA, USA). Library preparation was performed using Nextera DNA Flex Library Prep Kit according to the manufacturer’s instructions (Illumina Inc., San Diego, CA, USA). All isolates were subjected to WGS on an Illumina MiSeq platform (Illumina Inc., San Diego, CA, USA) at the Next Generation Sequencing Platform, Institute of Genetics, University of Bern. The software SeqSphere+ (version 7, Ridom GmbH, Münster, Germany) was used for the determination of ST, CT, *in silico* serotype, as well as the identification of resistance and virulence genes. The sequences were also screened for acquired resistance genes using the databases ResFinder (https://bitbucket.org/genomicepidemiology/resfinder_db.git, accessed 14 October 2021) and for resistance-associated chromosomal mutations using PointFinder (https://bitbucket.org/genomicepidemiology/pointfinder_db.git, accessed 22 April 2022). Determination of plasmid incompatibility groups was conducted by *in silico* replicon typing using the PlasmidFinder database (https://bitbucket.org/genomicepidemiology/plasmidfinder_db.git, accessed 20 March 2022).

The sequence data set was first inspected for within-sample (same calf and time) replicate isolates based on identical CT, *in silico* serotype, AMR genes, virulence genes, and plasmid incompatibility groups. Replicates were removed from the data set in order to prevent the overall strain diversity from being affected by the within-sample dominance of a strain. A phylogenetic tree of the remaining 155 isolates was generated using Ridom SeqSphere+ (version 8.5.1, Ridom GmbH, Münster, Germany) and visualized and annotated using iTOL (version 6.7.5, https://itol.embl.de). The data set was then inspected for clonal isolates within calves over time, as well as between calves. Isolates were defined as clones based on identical CT, whereas identical clones were additionally defined based on AMR genes and virulence genes. Replicates of identical clones were removed to create a data set of unique strains to prevent a disproportionate weight of clonal strains on the reported distribution of AMR determinants. When clonal isolates carried combinations of AMR determinants that differed from one another within a group (sharing the same CT), differences in plasmid incompatibility groups were inspected.

## Data Availability

The Illumina raw reads were deposited into the Sequence Read Archive (SRA) database of the National Center for Biotechnology Information (NCBI) under BioProject accession number PRJNA1005565 with BioSample accession numbers SAMN36988384 to SAMN36988647.

## SUPPLEMENTAL MATERIAL

Supplemental Table S1 spectrum.03214-23-s0001.xlsx Genome sequences of 264 *Escherichia coli* isolated from 22 calves.

ASM does not own the copyrights to Supplemental Material that may be linked to, or accessed through, an article. The authors have granted ASM a non-exclusive, world-wide license to publish the Supplemental Material files. Please contact the corresponding author directly for reuse.
